# Nutritional and Therapeutic Potential of *Cassia fistula* Pods: A Comprehensive Study on Mineral Composition, Immunomodulation, Antidiabetic Benefits, and Safety of Amaltas Tea in Southeast Punjab, Pakistan

**DOI:** 10.1002/fsn3.70752

**Published:** 2025-08-06

**Authors:** Muhammad Naeem Zubairi, Muhammad Khurram Afzal, Muhammad Tauseef Sultan, Mohammad S. Mubarak, Shaikh Jamal Uddin

**Affiliations:** ^1^ Department of Food Science and Technology, Faculty Food Science and Nutrition Bahauddin Zakariya University Multan Pakistan; ^2^ Deparment of Human Nutrition, Faculty Food Science and Nutrition Bahauddin Zakariya University Multan Pakistan; ^3^ Department of Chemistry The University of Jordan Amman Jordan; ^4^ Pharmacy Discipline, Life Science School Khulna University Khulna Bangladesh

**Keywords:** anti‐diabetic, anti‐enzyme, brine shrimp lethality, *Cassia fistula*, functional tea, immunomodulatory, mineral

## Abstract

*Cassia fistula,* a plant belonging to the Leguminosae family, grows in various regions, including South Africa, Asia, China, America, Canada, Bangladesh, Afghanistan, Pakistan, Brazil, and West India. Southeast Punjab, Pakistan, has long utilized *C. fistula* tea, which is becoming more popular due to its high mineral content, phytochemical compounds, and possible medical properties. This study investigates how to improve immunity, control diabetes, and ensure that it is safe to use. Knowing its makeup and effects might help justify its use in functional tea. The pods of *C. fistula* have been found to possess antidiabetic, anti‐enzyme, and immunomodulatory properties, as demonstrated by various tests. This work focuses on assessing properties and mineral profile analysis using the Association of Official Analytical Chemists standard protocol. The brine shrimp lethality method was employed to evaluate *C. fistula* pod‐based functional toxicity. Results of mineral contents demonstrated that copper (0.08 ± 0.03 ppm), iron (0.44 ± 0.01 ppm), manganese (0.14 ± 0.01 ppm), and zinc (0.27 ± 0.01 ppm) are present, enhancing the nutritional values of the pods. The functional tea from *C. fistula* pods showed the highest percentage of inhibitory activity against *α*‐glucuronidase, with a percentage of 60.66%, followed by *β*‐glucuronidase, *α*‐glucosidase, and *α*‐tyrosinase. According to immunomodulatory and toxicity studies, animals treated with *C. fistula* pod‐based functional tea showed lower blood glucose levels, increased serum antibody titer, and safety for human health. *C. fistula* pods, rich in minerals, have potential nutritional and therapeutic benefits, making Amaltas tea a safe and effective natural remedy in Southeast Punjab.

## Graphical abstract

Functional tea from *C. fistula* 's pod possesses a good amount of minerals, including copper, iron, manganese, and zinc in non‐toxic concentrations, and showed the highest percentage of inhibitory activity against α‐glucuronidase. It also further showed lower blood glucose than the control group in an animal model with higher immunomodulatory activity and no toxicity.

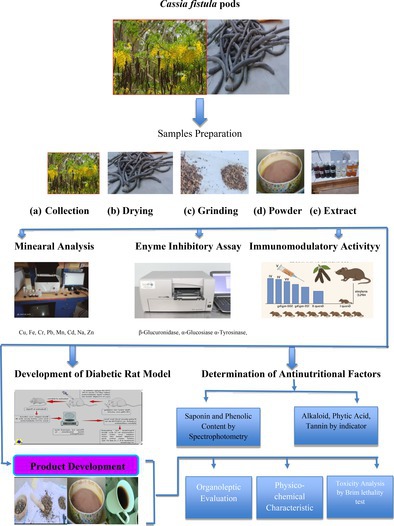

## 1 Introduction

Medicinal plants have gained significant attention due to their nutritional, therapeutic, and chemical composition, with 80% of people worldwide using them for food, fodder, and timber (Camo et al. 2021). Southeast Asian countries, including China, India, Pakistan, and Nepal, are the largest consumers (Afzal et al. 2021). Traditional and alternative medicine systems in Pakistan, particularly in the Cholistan and Pothohar regions, have flourished over generations (Akinyede and Amoo 2021). Medicinal plants, rich in phytochemical compounds, exhibit therapeutic potential for metabolic disorders, cancer, diabetes, and other ailments (Akiko et al. 2009). They also exert antioxidant and antiobesity properties, improving gut health and potentially delaying neurodegenerative disorders (Ahmad et al. 2021). The global trade of medicinal plants and herbal products is expected to reach 500 billion U.S. dollars by 2050 (Bazrafshan et al. 2015).

*Cassia fistula* is a medium‐sized deciduous tree in the Leguminosae family. It is known for its golden flowers and spreading branches (Tanveer et al. 2021). *C. fistula* pods are prominent in traditional medicine systems like Ayurveda for treating skin diseases, fever, and digestive disorders (Gavali et al. 2019). In modern pharmacology, it has drawn attention for its antidiabetic and immunomodulatory properties (Cohen 2022). Research findings indicate that its bioactive compounds, such as flavonoids and anthraquinones, may help regulate blood glucose and modulate immune responses. The demand for herbal treatments is increasing, leading to the exploration of new plant sources with medicinal properties (Orwa et al. 2019). Several in vivo and in vitro studies support its potential therapeutic role in managing diabetes and enhancing immunity. However, the pods' comprehensive nutritional and therapeutic potential, particularly in tea, has not been extensively studied (Orwa et al. 2019). Integrating both traditional and modern insights highlights the plant's broad pharmacological relevance. The Southeast Punjab region of Pakistan has a rich biodiversity, with the climate and soil composition making it an ideal place for *C. fistula* to thrive (Akinyede and Amoo 2021).

*C. fistula* pods, rich in phytochemicals, bioactive substances, and pharmacological compounds, are essential in treating various diseases and disorders (Sharma et al. 2022). These pods contain anthraquinones, including rhein, sennosides, and aloe‐emodin, which are known to influence intestinal secretion and absorption, often leading to diarrhea (Danish et al. 2011). In Thailand, these pods are used in the production of laxative pills (Das et al. 2022). The active forms of anthraquinones, derived from the breakdown of glycosides by intestinal flora, exhibit varying laxative and purgative effects (Tabrez et al. 2021). The findings of this study may contribute to the understanding of *C. fistula* as a promising ingredient for the development of nutritionally enriched food products, particularly in Southeast Punjab, Pakistan (Sakulpanich et al. 2012). Based on the previous discussion, the aim of the present work was to comprehensively evaluate the nutritional and therapeutic potential of *C. fistula* pods in the form of Amaltas tea, with a specific focus on the Southeast Punjab region of Pakistan. The investigation includes a detailed analysis of the pods' mineral composition, their immunomodulatory and antidiabetic effects, and an assessment of their safety for regular consumption. Furthermore, the study explores the antibacterial, anti‐inflammatory, and enzymatic inhibitory properties of Amaltas tea, highlighting its potential as a functional food and natural health‐promoting beverage.

## 2 Materials and Methodology

### 2.1 Sample Collection

One hundred grams of completely dried *C. fistula* pods were collected from Bahauddin Zakariya University, in the vicinity of Multan, Pakistan, during June 2022 and dried under the shade. Dr. Kausar Hussain Shah, a botanist at the Department of Botany, Bahauddin Zakariya University of Multan, Pakistan, identified and authenticated the plant (voucher specimen PCF01). A voucher specimen (PCF01) was deposited at the herbarium located at the Department of Botany, Bahauddin Zakariya University of Multan, Pakistan.

### 2.2 Mineral Analysis

The *C. fistula* pods were collected, thoroughly cleaned with tap water, and air‐dried under shade to preserve bioactive compounds. The dried pods were ground into a fine powder using a laboratory‐grade grinder, ensuring uniform particle size (< 1 mm), and stored in sealed, labeled polythene bags at room temperature (25°C) for further research. The extraction process followed the method outlined by Anitha and Miruthula (2020). Briefly, the powdered samples were soaked in an appropriate solvent (e.g., ethanol or methanol) in a 1:10 (w/v) ratio. The mixture was incubated overnight at 25°C and kept in a dark room for 48 h to prevent degradation of sensitive compounds. The extract was filtered through Whatman No. 1 filter paper and muslin fabric to separate the residue. The filtrates were transferred to labeled test tubes for further analysis. Mineral content and heavy metal concentrations were determined at the Soil Science Laboratory of Bahauddin Zakariya University, using the AOAC (1990) standard protocol. For digestion, 0.5 g of the powdered sample was treated with a digestion mixture containing concentrated sulfuric acid, perchloric acid, and nitric acid (2:1:1, v/v). The sample was incubated at 150°C until a clear solution was obtained. The digested sample was then diluted with deionized water to a final volume of 50 mL. The minerals analyzed included calcium, cadmium, chromium, copper, iron, lead, magnesium, manganese, potassium, sodium, and zinc. The analysis was performed using a PerkinElmer atomic absorption spectrophotometer (AAS) (Model No. HVO‐5310, American Global Corporation, USA) calibrated with certified reference materials to ensure accuracy. Quality controls, including blanks and standard solutions, were incorporated for validation. The detection limits for heavy metals such as cadmium, lead, and chromium were established based on AOAC guidelines.

### 2.3 Enzyme Inhibitory Assay

The *β*‐glucuronidase activity was determined by spectrophotometry using an optical absorbance measurement of *p*‐nitrophenol, formed from the substrate. Briefly, the reaction mixture made in a final volume of 100 μL contained 70 μL of 0.1 M acetate buffer pH 5.0 (3.402 g/250 mL of sodium acetate, pH was adjusted with 0.1 M acetic acid), 10 μL of test compound (0.5 mM), 10 μL of enzyme (24 U) pre‐incubated for 10 min, and 10 μL of 0.5 mM *p*‐nitrophenyl‐β‐D‐glucuronide (PNPG). The mixture was then incubated at 37°C (38°C ± 0.2°C) for 30 min (30 ± 0.2 min). The reaction was stopped by adding 50 μL of 0.2 M Na_2_CO_3_. After the reaction was terminated, the optical absorbance was measured against the reagent blank at 405 nm, using a Synergy HT BioTek 96‐well microplate reader. L‐aspartic acid was used as a positive control (Collins et al. 2019). The α‐glucosidase inhibitory activity was performed following the previous method described by Palanisamy et al. (2011), with some modifications. Plant extract (50 μL) was mixed with glutathione (50 μL), α‐glucosidase solution (50 μL) in phosphate buffer (pH 6.8), and PNPG (50 μL) in a 96‐well microplate and incubated for 15 min at 37°C. The reaction was stopped by adding 0.2 M sodium carbonate (50 μL), and the absorbance was measured at 400 nm (Tiwari et al. 2021). The α‐tyrosinase inhibitory activity was determined using the modified dopachrome method with slight modifications. Plant extract (25 μL) was mixed with tyrosinase solution (40 μL) and phosphate buffer (pH 6.8) (100 μL) in a 96‐well microplate and incubated for 15 min at 37°C. L‐3,4‐dihydroxyphenylalanine (L‐DOPA) (40 μL) was added to the mixture to initiate the reaction. The absorbance was read at 492 nm after 10 min incubation at 37°C (Lee et al. 2019).

The percentage of *β*‐glucuronidase, α‐glucosidase, and α–tyrosinase inhibition was calculated using the following equation:

$$\%\text{Inhibition}=\left[\left({\mathrm{Abs}}_{\text{blank}}\;-\;{\mathrm{Abs}}_{\text{sample}}\right)/{\mathrm{Abs}}_{\text{blank}}\right]\times 100$$

where Abs_blank_ is the absorbance of the blank and Abs_sample_ is the absorbance of the sample. Acarbose is used as a positive control, and IC_50_ was determined.

### 2.4 Immunomodulatory Activity

The immunomodulatory effect of the extract from the pods of *C. fistula* was examined using the method outlined by Laxmi et al. (2015). The extract was prepared from pods dried in the shade and coarsely powdered. The dried powder was extracted with triple‐distilled water using a Soxhlet apparatus. The extracts obtained were evaporated to dryness at 45°C using a hot air oven and then weighed. In this study, we examined *C. fistula* *'s* pod extract's immunomodulatory effect on rats' humoral immune systems. The extract was prepared by pulverizing the pod into a coarse powder and drying it at 45°C in a hot air oven for 10–15 min. The humoral immune response was assessed by measuring the antibody generated in various groups of rats given varying dosages of ‘O’ antigen and *C. fistula* 's pod extract. The *Salmonella typhimurium* ‘O’ antigen was prepared using conventional methods, with smooth *S. typhimurium* colonies developed on the Tryptose agar medium being injected into them. The infected soup was centrifuged for twenty minutes at 3000 rpm, washed with regular saline, and cooked for 2.5 h at 100°C after discarding the supernatant. This heated culture was used as the ‘O’ antigen to determine the humoral immune response in albino rats (Tiwary and Goel 2018). Rats were randomly divided into seven groups with five animals each. The first group was used as a negative control. For 21 days, the rats in groups V, VI, and VII were orally given 125 mg/kg, 250 mg/kg, and 500 mg/kg of pods (Bhatia et al. 2017). A serum agglutination test was used to evaluate the antibody titer in rats against *Salmonella* one week following the last dosage of the ‘O’ antigen. HPLC analysis of both extracts was employed to evaluate the presence of active immunomodulatory pods. An investigation on the effects of functional tea derived from *C. fistula* pods on albino rats exposed to 1‐chloro‐2, 4‐dinitrobenzene was conducted. The rats in Group I received DNCB and no plant extract, while those in Groups II, III, IV, V, VI, and VII received an extract from the pods (Cruickshank et al. 2018).

### 2.5 Development of Diabetic Rat Model

Our investigation involved 90 rats fed an unlimited high‐fat diet (HFD) for 2 weeks, followed by a single dose of streptozotocin (25 mg/kg) on day 14. The rats (FPH‐AEC‐2024‐123) with type I diabetes mellitus were exposed to this high dose, which did not destroy all cells. Diabetic rats with blood sugar levels less than 200 mg/dl were used in the experiment. After a 4‐day treatment with streptozotocin (STZ), the occurrence of diabetes was assessed by measuring fasting plasma glucose using tail vein blood samples. Hyperglycemia was defined as high blood glucose levels after 72 h. For 30 days, group II HFD + STZ‐induced diabetic rats received the *C. fistula* once daily at a dose of 0.45g/kg body weight (Garba et al. 2015). The *n*‐hexane extract from the pods of *C. fistula* was produced and standardized in the Food Science and Technology labs of Bahauddin Zakariya University in Multan, Pakistan. The rats were randomly divided into two groups: Group I received 1 mL of a 0.3% carboxymethyl cellulose solution daily along with regular food and unrestricted access to water, whereas Group II received oral tubes containing 0.3% carboxymethyl cellulose solution and 0.45 g/kg of *C. fistula* extract every day for 30 days (Atangwho et al. 2010). Blood samples were extracted intracardially after the thirty‐first day of the trial and analyzed using the glucose oxidase technique. Statistical analysis was performed using the (‘*t*’) test to determine the statistical significance of variable differences between the two groups. A *p*‐value of less than 0.05 indicated significance, while high significance was shown by a *p*‐value of less than 0.01 (Nirmala et al. 2008).

### 2.6 Determination of Antinutritional Factors

The antinutritional components in *C. fistula* pods were identified using various methods. The saponin content was determined using the McEwan et al. (2014) method, which involved combining a 10 g sample of powdered pods with acetone and then extracting it again for another day. Vanillin was used for color development with ethanol and H_2_SO_4_. The material was then placed through a spectrophotometer to determine saponin absorption at 500 nm. The alkaloid content was determined using the McEwan et al. (2014) method, which involved placing 1 mL of *C. fistula* pods in 10% acetic acid, followed by ethanol, and covering it for a day. The sample was then filtered and concentrated, and the residual alkaloid was dried and weighed. Phytic acid was determined using the Algadi and Yousif (2015) technique, which involved obtaining an extract from 2 g of powdered pods and 50 mL of 3% trichloroacetic acid (TCA) in a sample flask. The sample was heated in a boiling water bath, then rinsed with 3% TCA and distilled water, and then heated in a water bath at boiling for 30 min. The precipitate was washed in hot water (45°C) before being drained, filtered using filter paper, and diluted with distilled water.

The ferric ion concentration was estimated using the standard curve of various Fe(NO_3_)_3_ concentrations. At the same time, the tannin content was examined using a modified version of Algadi and Yousif's modified vanillin‐HCl in methanol technique. The results of tannic acid were replicated and expressed using a standard curve, such as the quantity of tannic acid (mg per mL) that produces color intensity comparable to that of tannin. The total phenol content was determined using Algadi and Yousif's (2015) technique, which involved mixing 3 mL of methanol in a 50 mL flask with 60 mg of powdered *C. fistula* pods and gently mechanically shaking the mixture. The resulting filtrate was mixed with 50 mL of distilled water, followed by adding 3 mL of 0.1 M FeCl_3_ in 0.1 N HCl and 3 mL of 0.008 M K_3_Fe(CN)_6_. The sample was measured using a spectrophotometer at 720 nm after a 10‐min interval. Preparing the tannic acid standard curve involved dissolving 100 mg of tannic acid in distilled water, making concentrations such as 0, 2, 4, 6, 8, and 10. The standard curve was established by plotting concentration against the corresponding absorbance value, demonstrating a linear correlation. The percentage of total polyphenols was calculated using the formula:

(1) $$\text{Percent total polyphenol}=\left(C\times 56\times 100\right)/60$$

(2) $$\text{Phytate}\;\left(\mathrm{mg}/100\;g\right)=\left(6/\text{4A}\times C\times 20\times 10\times 50\times 100\right)/\left(1000\times S\right)$$

(3) $$\mathrm{TC}\;\left(\%\right)=\left(C\times 10\times 100\right)/200$$

In Formula (1), 56 represents the total volume, and 60 is the weight of the sample (mg). In Formula (2), C represents the concentration corresponding to optical density, A represents optical density, and S represents the weight of the sample. In Formula (3), C denotes the concentration corresponding to the optical density, while 10 represents the total extract volume (mL), and 200 represents the sample weight (mg).

### 2.7 Product Development

Making functional tea from *C. fistula* pods involves manual harvesting and additional processing steps by following the method of Rabeta and Lai. Green pods are manually harvested and undergo withering to achieve 50%–55% moisture content. Rolling the dried pods causes the tissue to twist and break, releasing the liquid. Fermentation is then performed by spreading the traditional “dhool” in thin layers and exposing it to oxidation for 1–2 h at 25°C–30°C and 95% relative humidity. After fermentation, the “dhool” is heated to a final moisture content of 2%–3% in a tea dryer using hot air at 105°C. Green leaves and buds are chosen for functional tea. Microwave heating is used for 3 min, followed by dehydration at 65°C–75°C to attain 45% moisture. Well‐twisted green leaves are produced by rolling with a traditional piezy roller for 20 min at various pressures. The dried leaves have a final moisture content of 5%–6% after heating with hot air at 95°C–100°C in a dryer.

#### 2.7.1 Organoleptic Evaluation Study of *C. fistula* Pod‐Based Functional Tea

A panel of 10 judges (ORIC‐BZU/12345/2024) evaluated the sensory qualities of *C. fistula's* pod‐based functional tea using the 9‐point Hedonic Scale. The judges were chosen based on their ability to scale and distinguish across different qualities. The study's goals were explained to participants, and questionnaires were used to record their responses. Sensory tests were conducted in a room with minimal distractions, chemical and food odors, and sunshine (Akhtar et al. 2008).

#### 2.7.2 Physicochemical Characterization of *C. fistula* Pod‐Based Functional Tea

The weight of *C. fistula* pods was calculated using the AOAC (2000) methodology, with random mature pods weighed in grams per kilogram of fruit. Total soluble solids (%) in the pods were calculated using a hand refractometer 0%–32% TSS (Total Soluble Solid) at room temperature (25°C) and presented as percentage of TSS. The weight of pods was determined using an electronic balance and AOAC (2000) procedures.

#### 2.7.3 Chemical Characterization of *C. fistula's* Pod‐Based Functional Tea

We used Ranganna's (2014) technique to determine acidity, ascorbic acid, total carotenoids, total chlorophyll, protein, fat, fat percentage, and fiber content in functional tea made from *C. fistula* pods. Acidity was determined by macerating the material with distilled water, titrating against 0.1 N sodium hydroxide, and using 1% phenolphthalein solution as an indicator. Similarly, ascorbic acid was measured by macerating the sample with 3% meta‐phosphoric acid and titrating against 2,6‐dichlorophenol indophenol dye. In contrast, the total chlorophyll content was estimated using Rodriguez‐Amaya's (2004) approach, and protein was determined using the KELPLUS nitrogen estimation system and the micro‐Kjeldahl technique (AOAC 2005); fat percentage was calculated using Ranganna's technique and Soxhlet extraction, whereas the fiber percentage was estimated by dividing the decrease in weight of the sample by the sample weight and multiplying by 100. The study aimed to understand the properties of functional tea and its potential applications in various industries.

$$\%\mathrm{Fat}=\frac{\text{Total}\;\mathrm{wt}.\;\text{of}\;\mathrm{fat}}{\text{Sample weight}}\times 100$$

$$\%\text{Fiber}=\frac{\mathrm{Wt}.\;\text{of residue}-\mathrm{Wt}.\;\text{of}\;\mathrm{ash}}{\text{Total}\;\mathrm{wt}.\;\text{of sample}}\times 100$$

### 2.8 Toxicity Analysis of Functional Tea via Brine Shrimp Lethality Method

In this investigation, the toxicity of *C. fistula* pods functional tea was evaluated using a brine prawn mortality assay Iram et al. (2016). The bioassay aimed to evaluate the biological toxicity of functional tea formulations using brine shrimp ( *Artemia salina* ). We purchased dry eggs for the brine prawns from local stores in Multan, Pakistan, and mixed tap water with NaCl and baking soda to create faux seawater. After 48 h of incubation, the eggs were submerged in freshwater. This was followed by separately blending 300 L of functional tea, placing it in 96‐well plates, and drying it overnight. The toxins were then dissolved in 200 L of seawater containing 40–45 organisms. The final volume of the sample was incubated for 24–96 h at 26°C. The mortality rate was calculated by counting immobile larvae under a stereo microscope. The toxicity of each solution was determined in triplicate.

## 3 Results and Discussion

### 3.1 Mineral Analysis

The mineral analysis of *C. fistula* pods revealed the presence of significant amounts of Na, Fe, Cu, Cr, Pb, Mn, and Zn. The mineral content analysis of *C. fistula* pods indicates that they contain various essential and possibly hazardous elements in different quantities, as shown in Table 1. Copper (0.08 ± 0.03 ppm), iron (0.44 ± 0.01 ppm), manganese (0.14 ± 0.01 ppm), and zinc (0.27 ± 0.01 ppm) are present in non‐toxic concentrations and hence enhance the nutritional values of the pods. Among the measured minerals, sodium varied; the highest was 1.72 ± 0.1 ppm; sodium is very active in metabolism. The presence of lead at 0.06 ± 0.01 ppm and cadmium at 0.13 ± 0.02 ppm, even though very low, exceeded the safe limits and could pose health risks with long‐term consumption. In contrast, chromium was not detectable, reducing this metal's toxicity risk. Overall, the pods contain healthy minerals, but one must be cautious about minute quantities of lead and cadmium.

**TABLE 1:** *Cassia fistula* pod mineral contents.

Mineral content (ppm)	Symbol	Mean ± SD (ppm)
Copper	Cu	0.08 ± 0.03
Iron	Fe	0.44 ± 0.01
Chromium	Cr	ND
Lead	Pb	0.06 ± 0.01
Manganese	Mn	0.14 ± 0.01
Cadmium	Cd	0.13 ± 0.02
Sodium	Na	1.72 ± 0.1
Zinc	Zn	0.27 ± 0.01

*Note:* Values are the mean ± standard deviation (SD) of triplicate determinations.

### 3.2 Enzyme Inhibitory Assay

The percentage inhibition against *β*‐glucuronidase (IC_50_ = 60.66 ± 0.59), *α*‐glucosidase (IC_50_ = 10.29 ± 0.29), and *α*‐tyrosinase (IC_50_ = 76.98 ± 0.48) was observed in the *C. fistula* pod extract at 0.05 mg. In Table 2, the extract from *C. fistula* pods showed the highest percentage of inhibitory activity against *α*‐glucuronidase, with a percentage of 60.66%, followed by *β*‐glucuronidase, *α*‐glucosidase, and *α*‐tyrosinase. The half‐maximal inhibitory concentrations (IC_50_) of the extracts on the enzymes *β*‐glucuronidase, *α*‐glucosidase, and *α*‐tyrosinase were substantially higher than those of the control, L‐aspartic acid, acarbose, and kojic acid, respectively. In addition, extracts from *C. fistula* exhibited significantly higher potencies (*p* < 0.05) and higher levels of antiglucuronidase inhibitory action (IC_50_ = 33.80; 0.2 g/mL). Moreover, as shown in Table 2, there was no discernible variation in the antiglucuronidase activity of the extracts from the pods of *C. fistula* as compared to pure L‐aspartic acid. Furthermore, the present study also indicates that these IC_50_ values are significantly higher (*p* < 0.05) than those obtained with pure acarbose as an external control, suggesting that both extracts may be useful at doses higher than 0.05 mg for treating medical conditions, like T2D.

**TABLE 2:** *Cassia fistula* 's pod extract inhibitory effects on different polyphenol oxidase activities.

Extract	*β*‐Glucuronidase		*α*‐Glucosidase		*α*‐Tyrosinase	
	Inhibition% at 0.05 mg	IC_50_ (μg/mL)	Inhibition% at 0.05 mg	IC_50_ (μg/mL)	Inhibition% at 0.05 mg	IC_50_ (μg/mL)
*C. fistula*	60.66 ± 0.59^a^	33.8 ± 0.2^b^	10.29 ± 0.29^e^	55.31 ± 0.31^f^	76.98 ± 0.48^g^	39.2 ± 0.12
Control	—	33.8 ± 0.1^b^	—	38.61 ± 0.12	—	6 ± 0.01^i^
		L‐asp acid		Acarbose		Kojic acid

Based on three separate studies (*n* = 3), the data, including the IC_50_ values, are presented as the mean ± standard deviations (SD). Values with the same letter assigned to them suggest no significant difference (*p* > 0.05) between the enzymatic activity of the chemical control and another plant extract. Values denoted by unique letters in the statistical analysis of the extracts indicate a significant difference in the reported enzyme activity (*p* < 0.05). We are referring to the inhibitory concentration (IC_50_), which is 50.

### 3.3 Immunomodulatory Activity

#### 3.3.1 Induction of Humoral Immune Response

Results from this investigation demonstrated that, in comparison to control rats, all treated rats have higher antibody titers in serum samples. Furthermore, the results suggest that the serum antibody titer rose in the control group (115.33 ± 7) and in the *C. fistula* pod extract‐fed groups (152.33 ± 10, 260.0 ± 13, 263.0 ± 8), as shown in Table 3. This study confirms that there is a significant rise in antibody titer; thus, humoral immune response is induced in *C. fistula* pods fed rats, with a significantly higher immunomodulatory effect in *C. fistula* pod‐fed rats. The presence of quercetin in *C. fistula* pods might be responsible for the elevated humoral immune response, as quercetin has been reported to upregulate IFN‐γ & Th‐2 gene expression and production, which in turn modulate NK cell function and act as an immunostimulant (Nair et al. 2002).

**TABLE 3:** Humoral immune response: antibody titer increase.

Treatment	Nill	125 mg/kg	250 mg/kg	500 mg/kg
Control	115.33 ± 7	115.33 ± 7	115.33 ± 7	115.33 ± 7
Extract of pods	—	152.33 ± 10	260.0 ± 13	263 ± 8

### 3.4 Induction of Cell‐Mediated Immune

Results revealed that skin thickness improves noticeably at almost all extract dosages. The most significant increases in skin thickness in rats given 250 mg/kg body weight pods at 24, 48, and 72 h were 28.1%, 22.1%, and 15.5%, respectively. Furthermore, the results demonstrated that the amount of skin thickness increased in albino rats fed with 500 mg/kg body weight of pods was less than that of rats fed with 125 mg/kg body weight of either extract. Comparative analysis with parallel studies suggests that patients treated with *C. fistula* pulp showed a significant decrease in serum IgE level, producing significant relief in patients suffering from eczema or leprosy (Das et al. 2008). Similarly, several researchers have demonstrated the immune system modulation activities of *Cassia auriculata* & *Cassia tora* (Chakraborty 2009; Tilwari et al. 2011).

### 3.5 Development of Diabetic Rat Model

#### 3.5.1 Analysis of Antidiabetic Activity of *C. fistula* Pods

A randomized, controlled investigation was conducted on 90 rats. The effects of *C. fistula* pods on the blood levels of insulin and glucose in male albino rats with diabetes were compared and calculated. The serum glucose and insulin readings for the two groups are displayed in Table 4. Using a student's t‐test, it was found that the group II mean and standard deviation for insulin and blood glucose were significantly lower (*p* = 0.01, 0.02) than those of the control group (215.78 ± 4.6 mg/dL, 71.77 ± 3.7 μU/ml, and 208.631 ± 0.3 mg/dL and 66.77 ± 5.7 U/mL, respectively).

**TABLE 4:** A comparison of the diabetic experimental group's and control group's serum glucose and insulin levels.

Parameters	Group I	Group II	*p*
Serum glucose (mg/dL)	215.78 ± 4.6	208.63 ± 10.3	0.02*
Serum insulin (μU/mL)	71.77 ± 3.7	66.77 ± 5.7	0.01*

*Note:* The data are expressed as the mean ± SD, and a *p*‐value of less than 0.05 is deemed significant. The *p*‐values of the results of serum glucose and serum insulin are 0.02 and 0.01, respectively, less than *p* < 0.05, so that * indicates highly significant results.

### 3.6 Determination of Antinutrition Factors

The nutritional profile of *C. fistula* pods was assessed by measuring the quantity of antinutritional factors, as this is crucial in determining how nutritionally adequate they are. An antinutritional factor analysis revealed that the pods of *C. fistula* included phytic acid, total phenolic content, tannin, alkaloids, and saponin. Antinutritional chemical results were shown to be significant at *p* > 0.05. Table 5 displays the *C. fistula* pods' antinutritional characteristics, which show that the total phenolic content, tannin, alkaloids, and saponin mean reductions in *C. fistula* pods were 11.6%, 2.36%, 0.19%, and 0.14%, respectively. The mean decrease in phytic acid was 649.12 mg/100 g. The average reduction in total phenolic content was also greater than that of tannin, alkaloids, and saponin, while the average reduction of saponin was lower than that of alkaloids, tannin, and total phenolic content, as shown in Table 5. The findings demonstrate that *C. fistula* pods are crucial in reducing the antinutritional component that is listed in Table 5 and have a significant impact on this decrease.

**TABLE 5:** The level of antinutritional factors in *Cassia fistula* pods.

Antinutritional factors	Mean ± SD
Phytic Acid (mg/100 g)	649.12 ± 0.99
Total phenols (%)	11.6 ± 0.75
Tannin (%)	2.36 ± 0.02
Alkaloid (%)	0.19 ± 0.02
Saponin (%)	0.14 ± 0.01

The proportion over the values of the related *C. fistula* pods is shown by parentheses. The average of three values given on a dry weight basis made up each value. Means are values plus the standard deviation. According to Duncan's multiple range tests, means without a common letter in a column are significant at *p* > 0.05.

### 3.7 Product Development

#### 3.7.1 Sensory/Physical Evolution of *C. fistula* Pod‐Based Functional Tea

The sensory characteristics of goods, such as functional tea, are crucial for studying the potential use of herbs. Table 6 lists the sensory evaluation of functional tea with *C. fistula* pods prepared at three different concentrations for color, taste, texture, flavor, mouthfeel, and overall acceptability (OA). Color and flavor were liked most in control tea (T0), with scores of 7.57 ± 0.58 and 8.15 ± 0.50, respectively. The taste and overall acceptability were liked best in the 25% *C. fistula* pod tea, T5, with scores of 7.92 ± 0.38 and 8.30 ± 0.40, respectively. Scores for 10% and 15% *C. fistula* pods tea, T1 and T2, respectively, achieved high scores in taste and texture but were inferior to T5 regarding overall acceptability. The color and overall acceptability of T4 (20% *C. fistula* pods tea) were lower when compared to T5; however, the texture and mouthfeel were rated as very good. The acceptability of 25% *C. fistula* pods tea was generally more inconsistent at higher concentrations. This reveals that even though higher concentrations of *C. fistula* pods may have some detrimental effects on the sensory attributes, they enhance others that are desirable in flavor and taste.

**TABLE 6:** Sensory evaluation of *Cassia fistula* pod‐based functional tea.

T	Color	Taste	Texture	Flavor	Mouthfeel	OA
T_o_	7.57 ± 0.58^AB^	8.80 ± 1.04^AB^	5.50 ± 1.32^AB^	8.15 ± 0.5^B^	9.61 ± 0.53^AB^	8.19 ± 0.56^AB^
T_1_	6.09 ± 0.09^C^	9.80 ± 0.32^AB^	6.45 ± 0.87^AB^	8.43 ± 0.9^B^	8.5 ± 0.5^B^	8.22 ± 0.26^AB^
T_2_	5.47 ± 0.51^B^	8.92 ± 0.38^A^	5.83 ± 0.76^A^	8.35 ± 0.66^A^	9.67 ± 0.58^A^	8.35 ± 0.4^A^
T_3_	6.70 ± 0.67^A^	7.33 ± 1.15^B^	6.07 ± 1.26^B^	7.57 ± 0.58^C^	8.73 ± 0.64^C^	7.53 ± 0.57^B^
T_4_	4.57 ± 0.58^AB^	7.80 ± 1.04^AB^	6.50 ± 1.32A^B^	7.15 ± 0.5^B^	8.61 ± 0.53^AB^	8.10 ± 0.56^AB^
T_5_	8.47 ± 0.51^B^	7.92 ± 0.38^A^	7.83 ± 0.76^A^	8.15 ± 0.66^A^	9.27 ± 0.58^A^	8.30 ± 0.4^A^

*Note:* T_0_: Control, T_1_: 5% + 95% *Cassia fistula* pods + Water, T_2_: 10% + 90% *Cassia fistula* pods + Water, T_3_: 15% + 85% *Cassia fistula* pods + Water, T_4_: 20% + 80% *Cassia fistula* pods + Water, T_5_: 25% + 75% *Cassia fistula* pods + Water. The superscript letters (A, B, C, AB) indicate significant differences among treatments within each sensory attribute column, based on statistical comparison (e.g., ANOVA followed by post‐hoc test).

Abbreviations: OA, overall acceptability; T, treatment.

#### 3.7.2 Analysis of Physicochemical Characteristics of *C. fistula* Pod‐Based Functional Tea

Green tea, rich in phytochemicals like catechins, is known for its nutritional value and potential health benefits. It is used as a stimulant, digestive aid, and fever‐reducing agent (Floros et al. 2023). Functional tea contains antioxidants that protect the skin from UV damage, and its physicochemical characteristics have been studied (Abdelhakim et al. 2022). The physicochemical characteristics of the functional tea were investigated; results are shown in Table 7. Results indicated that the weight of pods in functional tea, total soluble solids, acidity, ascorbic acid, total carotenoids, total chlorophyll, fiber, fat, and protein are 1.5 g, 7.5%, 6.5%, 5 mg, 26 mg, 1.4 mg, 60%, 0%, and 0.1%, respectively.

**TABLE 7:** Physicochemical and functional characteristics of functional tea.

Sr.#	Parameters	*Cassia fistula* pod‐based functional tea
1	Weight of pods in functional tea (g)	1.5 ± 0.1
2	Total soluble solids (%)	7.5 ± 0.3
3	Acidity (%)	6.5 ± 0.1
4	Ascorbic acid (mg/100 g or mL)	5 ± 0.2
5	Total carotenoids (mg/100 g or ml)	26 ± 0.74
6	Total chlorophyll (g/L)	1.4 ± 0.05
7	Fiber (%)	60 ± 0.6
8	Fat (%)	0.0 ± 0.0
9	Protein (%)	0.1 ± 0.01

### 3.8 Toxicity Analysis of Functional Tea via Brine Shrimp Lethality Method

The products (functional tea prepared from the pods of *C. fistula* ) are harmless, as confirmed by the bioassay of brine prawns ( *A. salina* ). To confirm the safety of functional tea obtained from *C. fistula* pods, a bioassay employing brine shrimp ( *A. salina* ) was used to assess the biological toxicity of both treated and untreated tea. The mortality response of the newly born prawn larvae with treated and untreated products was reported at a temperature of 26°C and time intervals of 24**–**96 h. The blank seawater used to compute natural mortalities is treated only with solvent. The percentage of mortality is calculated using the average number of dead larvae/concentration. The highest death rates at 96 h were observed in prawns exposed to methanol (17.5%, 97.5%, and 20%, respectively). Another aspect of the study was to evaluate the breakdown of a poisonous component in a medicinal plant, pomegranate. The samples were obtained from the plant expert at Bahauddin Zakariya University in Multan, Pakistan's Faculty of Agricultural Sciences and Technology. Methanolic extracts at a concentration of 1000 μg/mL showed the maximum mortality, with a larvicidal activity of 30%. In contrast, at the same dose, the larval death rates of the methanolic, acetone, and water extracts were 20%, 16.7%, and 3.3%, respectively; these results are listed in Table 8.

**TABLE 8:** Toxicity of the product (functional tea) analyzed via brine shrimp lethality.

Treatments	Incubation period (h)	Living shrimps (No.)	Dead shrimps (No.)	Mortality %
Control				
Sea water ± shrimps	24	40	0	0
	48	40	0	0
	72	40	0	0
	96	38	2	5
Methanol ± shrimp	24	38	2	5
	48	39	1	2.5
	72	34	6	15
	96	33	7	17.5
Untreated toxins ± shrimp	24	8	32	80
	48	5	35	87.5
	72	2	38	95
	96	1	39	97.5

Treatments	Incubation period (h)	Living shrimps (No.)	Dead shrimps (No.)	% Mortality
Treated toxin with functional tea extract of *Cassia fistula pods ±* shrimps	24	39	1	2.5
	48	38	2	5
	72	34	6	15
	96	30	10	25

## 4 Discussion

The *C. fistula* (Amaltas) pods have long been used in traditional medicine for their therapeutic properties, and recent studies have validated their potential, particularly in the form of functional tea. Tea, rich in essential minerals like iron, zinc, and sodium, supports metabolic processes and immune health. Its significant immunomodulatory effects, enhancing both humoral and cell‐mediated immune responses, make it a promising natural remedy for strengthening the immune system. Moreover, *C. fistula* pod extracts have shown antidiabetic properties, effectively lowering blood glucose and insulin levels. This suggests its use in managing type 2 diabetes, especially in areas with limited access to modern treatments. The tea's bioactive compounds also inhibit enzymes like β‐glucuronidase and α‐glucosidase, managing inflammatory conditions, skin diseases like psoriasis, and carbohydrate metabolism, potentially reducing the risk of diabetes and cardiovascular diseases. Toxicity studies confirm that *C. fistula* tea is safe for consumption, offering a natural, non‐toxic option for managing various health conditions.

Mineral analysis of *C. fistula* pods demonstrated the presence of essential minerals such as sodium (Na), iron (Fe), copper (Cu), lead (Pb), manganese (Mn), cadmium (Cd), and zinc (Zn). Sodium, at 1.7 ppm, plays a critical role in metabolite transport and the prevention of arteriosclerosis and hypertension (Adeyeye and Otokiti 1999); this aligns with its recognized function in regulating blood pressure (Zayed and Terry 2003). The iron content, measured at 0.44 ppm, is below the FAO/WHO permissible limits set in 1984 (Hussain et al. 2009), suggesting that *C. fistula* pods may contribute positively to nutritional health, particularly in lipid, protein, and carbohydrate oxidation and central nervous system (CNS) regulation. Copper and zinc levels, at 0.08 and 0.27 ppm, respectively, are also within safe limits and below FAO/WHO standards (Heyes 1997). Interestingly, no chromium (Cr) was detected, reducing potential risks related to kidney, liver, or lung damage. However, low concentrations of lead and cadmium, at 0.06 and 0.13 ppm, respectively, are still above recommended limits, posing a potential long‐term health risk (Hussain et al. 2009). In contrast, zinc deficiency is a global concern, particularly in China, India, the U.S., and the U.K., affecting 20% of the population and underscoring the importance of zinc supplementation (Gupta et al. 2020). On the other hand, sodium (Na) is essential for nerve function, muscle contraction, and fluid balance. Unlike other metals discussed, sodium's physiological importance and potential health risks from excessive intake, such as hypertension, are not sufficiently emphasized. While iron (Fe), zinc (Zn), and copper (Cu) are crucial for enzymatic and metabolic functions, toxic metals like lead (Pb) and cadmium (Cd) pose serious health threats even at low concentrations. Manganese (Mn) is essential but can be neurotoxic in excess. A balanced inclusion of sodium analysis would provide a more comprehensive health impact assessment of these elements.

In this work, we have explored the enzyme inhibitory activities of *C. fistula* pods. At a concentration of 0.05 mg, the extract showed significant inhibitory activity against key enzymes like β‐glucuronidase, α‐glucosidase, and α‐tyrosinase. These enzymes are critical in metabolic and dermatological processes, making the pods valuable for pharmaceutical, food, and cosmetic industries (Koehler 1979). The extract exhibited a 60.66% inhibition of β‐glucuronidase, placing *C. fistula* as a promising natural treatment for enzyme‐related conditions. Additionally, the inhibition of α‐glucosidase, a key enzyme in carbohydrate metabolism, indicates the potential use of the pods in managing type 2 diabetes. The IC_50_ values of the extracts were comparable to pharmaceutical agents like L‐aspartic acid and acarbose, suggesting that *C. fistula* contains bioactive compounds with therapeutic potential (Hirsh et al. 1997). The inhibition of α‐tyrosinase suggests the potential use of the pods in skincare, particularly for preventing hyperpigmentation and promoting skin whitening (Zengin et al. 2015; Gulati et al. 2012). Research findings have shown that bioactive compounds in *C. fistula* can prevent skin damage or premature aging, making them valuable ingredients in cosmetics (Holtzinger 1996; Declercq et al. 2009).

Immunomodulatory studies on *C. fistula* pods demonstrated significant enhancement in humoral immune responses, as indicated by increased antibody titers in rats. The highest titers were observed in the group administered 500 mg/kg of the extract. Quercetin dihydrate, a bioactive compound identified in the pods, is known to upregulate immune‐related gene expression and stimulate NK cell activity, further supporting the hypothesis that *C. fistula* pods act as natural immunostimulants (Nair et al. 2002). Kaempferol, another bioactive compound in the pods, has been linked to enhanced T‐lymphocyte proliferation and cytokine production, contributing to the immune response (Swarnalatha and Puratchikody 2014). The immunomodulatory effects of the pods suggest their potential as therapeutic agents in boosting immune defenses (Toit et al. 2009). Chakraborty (2009) reported similar immune‐modulating properties in *Cassia tora* and *Cassia auriculata* , further confirming the medicinal potential of this genus.

A separate study on cell‐mediated immunity showed that *C. fistula* pod extracts significantly increase skin thickness in rats, a marker of immune response activation. This enhancement was dose‐dependent, with the highest effects observed at 250 mg/kg body weight. These findings suggest potential applications in treating skin disorders such as dermatitis and leprosy (Declercq et al. 2009). The immune‐enhancing effects observed were attributed to the pods' bioactive compounds, which boost both humoral and cell‐mediated immune responses. In diabetic rat models, *C. fistula* pods exhibited significant antidiabetic activity, showing reductions in serum glucose and insulin levels, comparable to standard antidiabetic medications like metformin. The hexane extract of *C. fistula* pods, at a dose of 0.45 g/kg, produced notable hypoglycemic effects, making it a potential remedy for managing type 2 diabetes. These findings align with previous studies by Nirmala et al. (2008) and Prabhakar and Doble (2011), who observed similar reductions in blood glucose levels and insulin resistance using *C. fistula* bark extracts. Studies by Mwangi et al. (2021) and Garba et al. (2015) further confirmed the pods' ability to lower blood sugar in diabetic rats, reinforcing the plant's consistent antidiabetic properties across different parts.

Analysis of the antinutritional factors in *C. fistula* pods revealed the presence of compounds like phytic acid, tannins, alkaloids, and saponins, which can impact nutrient absorption. Phytic acid, at 649.12 mg/100 g, was the most prevalent antinutritional factor, followed by total phenolic content (11.6%), tannins (2.36%), alkaloids (0.19%), and saponins (0.14%). These levels are consistent with other findings, such as those reported by Osman (2004) and Algadi and Yousif (2015). While these compounds can reduce nutrient bioavailability, the overall nutritional value of the pods remains positive due to their rich mineral content. Product development research on functional tea made from *C. fistula* pods showed promising sensory and physicochemical properties. Sensory evaluations confirmed that the tea was well accepted, with the highest flavor and overall acceptability scores at a 15% pod concentration. This suggests that *C. fistula* pods have potential in the functional food sector, mainly due to their high antioxidant content and protective effects against UV damage (Arivuchudar and Nazni 2020; Rezapour et al. 2016). The addition of *C. fistula* to functional tea formulations improved sensory qualities like flavor, texture, and overall acceptability (Pavlova and Nakov 2019), supporting its use as a fortification tool in food products (Bazrafshan et al. 2015).

Finally, toxicity assessments using the brine shrimp lethality method confirmed the safety of *C. fistula* pod extracts. Mortality rates in shrimp exposed to tea were significantly lower than those in control samples, indicating that the tea is non‐toxic and safe for human consumption (Iram et al. 2016). This supports the potential for *C. fistula* pods to be used in functional food products without adverse health effects. Overall, *C. fistula* pods offer significant promise in various therapeutic, nutritional, and functional applications. Their rich mineral content, immune‐boosting and antidiabetic properties, and low toxicity make them valuable for functional foods, pharmaceuticals, and cosmetics. The evidence from this study provides a strong foundation for the broader use of *C. fistula* in health and wellness products.

## 5 Conclusions

This comprehensive study confirms the therapeutic and nutritional potential of *C. fistula* pods. Our findings suggest that the pods are safe for human consumption at recommended dosages, as verified by brine shrimp lethality testing. No mortality was observed in test organisms, indicating low toxicity at appropriate levels. However, excessive intake of pod extract may be harmful, suggesting a need for dosage regulation. The pods demonstrated promising immunomodulatory properties in animal models. They also exhibited significant potential in managing type II diabetes. Phytochemical analysis revealed the presence of beneficial compounds like phenols, tannins, sterols, flavonoids, and phytic acid. These compounds contribute to the plant's antioxidant, antidiabetic, and immune‐boosting effects. In addition, our findings showed that antinutritional factors were present but within acceptable limits, posing no threat at safe dosages. Mineral analysis highlighted a rich profile, making the pods nutritionally valuable. The study supports the development of functional Amaltas tea using these pods. Short‐term use appears safe, but long‐term efficacy and safety require further research. This functional tea holds promise as an affordable, natural remedy in Southeast Punjab, Pakistan. Its ability to aid in diabetes management, boost immunity, and offer nutritional benefits is noteworthy. With further validation, *C. fistula* pods could become a valuable addition to food development, functional food, medicinal remedies, and dietary supplements.

## Author Contributions

**Muhammad Naeem Zubairi:** conceptualization (equal), methodology (equal), writing – original draft (equal). **Muhammad Khurram Afzal:** formal analysis (equal), supervision (equal), writing – original draft (equal), writing – review and editing (equal). **Muhammad Tauseef Sultan:** conceptualization (equal), data curation (equal), investigation (equal), methodology (equal), writing – review and editing (equal). **Mohammad S. Mubarak:** formal analysis (equal), investigation (equal), project administration (equal), resources (equal), validation (equal), visualization (equal). **Shaikh Jamal Uddin:** conceptualization (equal), data curation (equal), formal analysis (equal), investigation (equal), methodology (equal), visualization (equal), writing – original draft (equal), writing – review and editing (equal).

## Ethics Statement

The authors have nothing to report.

## Consent

The authors have nothing to report.

## Conflicts of Interest

The authors declare no conflicts of interest.

## Data Availability

The data that support the findings of this study are available on request from the corresponding author.
